# Mortality risk prediction in NSTE-ACS following PCI: Insights from a real-world cohort

**DOI:** 10.1371/journal.pone.0336130

**Published:** 2025-11-06

**Authors:** Shifa Geng, Yubao Luo

**Affiliations:** Department of Cardiology, Tangshan Hongci Hospital, Tangshan, Hebei, China; Medizinische Universitat Graz, AUSTRIA

## Abstract

**Background:**

Non–ST-segment elevation acute coronary syndrome (NSTE-ACS) is a major contributor to cardiovascular mortality, yet reliable tools for individualized mortality prediction remain limited. Machine learning offers the potential to enhance prognostic accuracy in this high-risk population.

**Methods:**

A total of 1,495 patients with NSTE-ACS who underwent percutaneous coronary intervention (PCI) were retrospectively analyzed. Eight clinical and laboratory variables were selected through univariate and multivariate logistic regression. Five machine learning models-logistic regression, random forest, XGBoost, LightGBM, and naïve Bayes-were constructed. Model performance was evaluated using area under the curve (AUC) and calibration curves.

**Results:**

Age, diabetes mellitus, and ejection fraction were identified as independent predictors of all-cause mortality. Among all models, LightGBM achieved the highest AUC (0.847), followed by XGBoost (0.822), both of which demonstrated superior discrimination and calibration compared to traditional logistic regression and other algorithms. Calibration analysis showed excellent agreement between predicted and observed mortality in both training and test cohorts.

**Conclusion:**

Gradient boosting models, particularly LightGBM and XGBoost, significantly improve mortality prediction in NSTE-ACS patients after PCI. These models may facilitate more accurate risk stratification and guide personalized post-procedural management strategies in clinical practice.

## 1. Introduction

Non–ST-segment elevation acute coronary syndrome (NSTE-ACS), which includes non–ST-elevation myocardial infarction (NSTEMI) and unstable angina, accounts for the majority of acute coronary syndrome (ACS) presentations and continues to be a leading cause of cardiovascular morbidity and mortality worldwide [1,2]. Despite the widespread use of guideline-directed medical therapy and percutaneous coronary intervention (PCI), risk stratification in NSTE-ACS remains a clinical challenge due to the heterogeneity of patient characteristics and varying responses to treatment [3,4].

All-cause mortality is a clinically meaningful and comprehensive endpoint that reflects the overall effectiveness of cardiovascular management. Patients with comorbidities such as diabetes mellitus, renal dysfunction, or reduced left ventricular ejection fraction are particularly vulnerable to adverse outcomes following PCI [5]. Existing clinical scoring systems, including the Global Registry of Acute Coronary Events (GRACE) and Thrombolysis in Myocardial Infarction (TIMI) risk scores, have been widely adopted to guide risk stratification in ACS populations [6,7]. However, these models rely on limited input variables, are based on linear assumptions, and may fail to capture complex interactions within large-scale real-world datasets.

In recent years, machine learning (ML) has emerged as a promising methodology in cardiovascular medicine for its ability to model nonlinear relationships, incorporate high-dimensional data, and improve predictive accuracy [8,9]. Supervised learning algorithms such as logistic regression, random forest, extreme gradient boosting (XGBoost), light gradient boosting machine (LightGBM), and naïve Bayes have been increasingly applied to risk prediction tasks across various cardiac conditions, including heart failure, myocardial infarction, and atrial fibrillation [10–13]. Compared to traditional regression-based methods, ML models offer flexibility in handling feature interactions, missing data, and variable importance estimation [14].

Several studies have demonstrated the feasibility of using ML approaches in ACS populations. For example, Ke et al. developed a random forest model to predict in-hospital mortality in ACS patients and achieved superior performance over conventional risk scores [15]. In another study, Kaveh et al. utilized SHAP methods to forecast major adverse cardiovascular events following PCI [16]. Nevertheless, few investigations have focused specifically on the NSTE-ACS subgroup, which comprises a clinically diverse and diagnostically complex population with unique risk profiles. Moreover, direct comparisons of multiple ML algorithms within the same NSTE-ACS cohort are limited, and external validation of such models remains rare.

To address these gaps, the present study aimed to develop and compare the performance of five supervised ML models including logistic regression, random forest, XGBoost, LightGBM, and naïve Bayes for predicting all-cause mortality in patients with NSTE-ACS who underwent PCI. Using a real-world cohort, we assessed model discrimination and calibration through receiver operating characteristic analysis and calibration plots. Our goal was to identify the most effective algorithm for individualized risk prediction in this high-risk population, thereby contributing to improved clinical decision-making.

## 2. Materials and methods

### 2.1. Study design and patient selection

The clinical data analyzed in this study were derived from a publicly available dataset (https://doi.org/10.5061/dryad.13d31) uploaded by Yao HM et al [17]. This retrospective study was approved by the Ethics Committee of the First Affiliated Hospital of Zhengzhou University, with a waiver of informed consent due to the anonymized and non-interventional nature of the data. The dataset used in the present study was derived from a previously published cohort described by Yao HM et al. In brief, a total of 2,533 consecutive patients with coronary artery disease (CAD) who underwent percutaneous coronary intervention (PCI) with drug-eluting stents (DES) between July 2009 and August 2011 were included. All patient data were de-identified prior to analysis, and no identifiable personal information was accessible to the authors. For the present analysis, a total of 1495 patients diagnosed with non-ST elevation acute coronary syndrome (NSTE-ACS) were selected. The inclusion criteria were: (1) patients who underwent percutaneous coronary intervention with drug-eluting stents between July 2009 and August 2011; and (2) patients diagnosed with NSTE-ACS. The exclusion criteria were: (1) patients with missing or inconsistent baseline clinical data (e.g., sex, BMI, glycemia, TG, HDL-C, age); and (2) patients without a confirmed diagnosis of NSTE-ACS. All patients received PCI based on standard procedural protocols. Antiplatelet pretreatment with aspirin (300 mg) and clopidogrel (300 mg) was administered unless patients had already been on antiplatelet therapy prior to the intervention. Medical management after discharge followed the contemporary clinical guidelines. Patients with missing key clinical variables or unclear outcomes were excluded to ensure the reliability of the analysis.

### 2.2. Data collection

Baseline clinical characteristics—including age, sex, body mass index (BMI), history of hypertension, diabetes mellitus (DM), smoking, heart failure (HF), atrial fibrillation (AF), old myocardial infarction (OMI), chronic obstructive pulmonary disease (COPD), stroke, and peripheral vascular disease (PVD)—were extracted from patients’ electronic medical records. Laboratory parameters such as fasting blood glucose (Glu), serum creatinine (Cr), uric acid (UA), total bilirubin (BIL), total cholesterol (TC), triglycerides (TG), high-density lipoprotein cholesterol (HDL-C), and low-density lipoprotein cholesterol (LDL-C), as well as cardiac function indicators such as ejection fraction (EF), were obtained from the hospital’s laboratory information system. In addition, information on antiplatelet medication use-including aspirin, clopidogrel, and statins-was also collected from medication administration records.

### 2.3. Endpoints and follow-up

The primary endpoint of this study was **all-cause mortality**. Follow-up data were collected through outpatient clinic visits, hospital readmissions, or structured telephone interviews. Mortality status was confirmed using hospital records and follow-up documentation to ensure accuracy. Follow-up after a median of 29.8 months (quartiles, 25.6–34 months).

### 2.4. Statistical analysis

Continuous variables were expressed as means ± standard deviations (SD) and compared using the student’s *t*-test or Mann–Whitney *U* test, as appropriate. Categorical variables were presented as counts and percentages, and comparisons between groups were performed using the chi-squared (χ²) test or Fisher’s exact test.

Univariate logistic regression was first conducted to identify potential predictors associated with all-cause mortality after PCI. Variables with *P* < 0.1 in the univariate analysis were included in the multivariate logistic regression model to determine independent risk factors. Odds ratios (ORs) and 95% confidence intervals (CIs) were reported. Multicollinearity was assessed using variance inflation factor (VIF), and a two-sided *P* value < 0.05 was considered statistically significant.

To enhance predictive performance, five machine learning algorithms were employed: logistic regression, random forest, XGBoost, LightGBM, and naive Bayes. Specifically, XGBoost is an open-source library originally developed by Tianqi Chen and collaborators (University of Washington), and LightGBM is an open-source framework developed and maintained by Microsoft Research. The dataset was randomly divided into training and test cohorts. Model development and hyperparameter tuning were conducted using 10-fold cross-validation in the training cohort. Performance metrics including the area under the receiver operating characteristic curve (AUC), accuracy, sensitivity, specificity, positive predictive value, and negative predictive value were calculated based on the test set. Model calibration was assessed using calibration curves. All statistical analyses were performed using R software (version 4.3.1), and machine learning modeling was implemented with appropriate R packages.

## 3. Results

### 3.1. Baseline clinical characteristics

**Fig 1:**
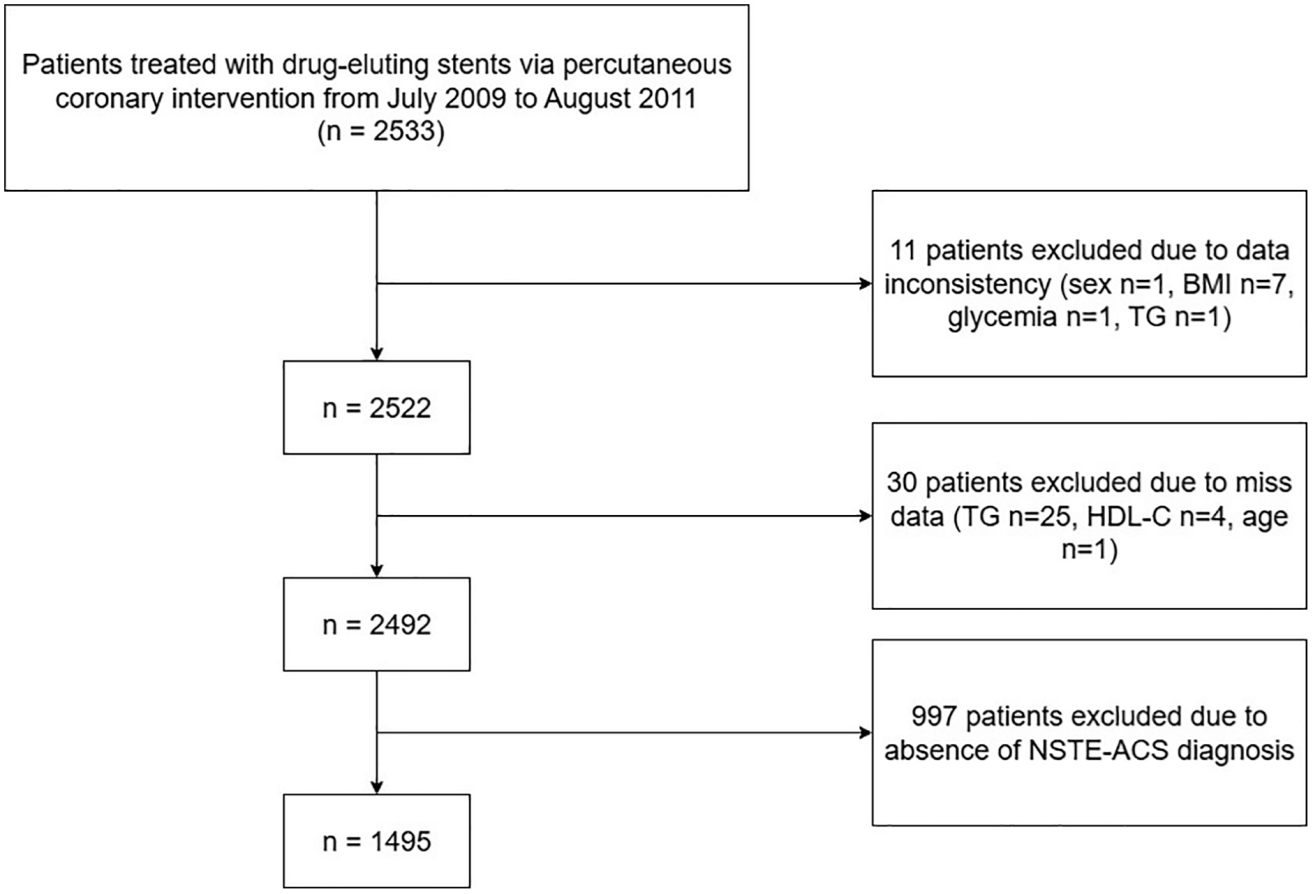
The flowchart of study participants.

As shown in Fig 1, a total of 1,495 patients diagnosed with NSTE-ACS and treated with PCI were included in this study. Based on all-cause mortality during follow-up, patients were classified into a death group (n = 108) and a survival group (n = 1,387). As shown in Table 1, there were significant differences in several baseline characteristics between the two groups. Patients who died were significantly older than those who survived (68.78 ± 10.10 vs. 59.87 ± 10.57 years, *P* < 0.001). The proportions of comorbidities including diabetes mellitus (37.0% vs. 20.3%, *P* < 0.001), heart failure (25.9% vs. 11.5%, *P* < 0.001), and old myocardial infarction (11.1% vs. 4.2%, *P* = 0.002) were notably higher in the death group. Other factors such as sex distribution, BMI, hypertension, smoking status, atrial fibrillation, COPD, stroke, and PVD did not differ significantly between groups (*P* > 0.05 for all).

**Table 1:** Baseline clinical characteristics of patients.

	Death (n = 108)	Survival (n = 1387)	Total (n = 1495)	P valuse
Age (years)	68.78 ± 10.10	59.87 ± 10.57	60.51 ± 10.78	<0.001
Male gender, n (%)	63 (58.3)	884 (63.7)	947 (63.3)	0.308
BMI (kg/m2)	24.68 ± 3.62	24.23 ± 3.77	24.26 ± 3.76	0.344
Hypertension, n (%)	63 (58.3)	763 (55.1)	826 (55.3)	0.575
DM, n (%)	40 (37.0)	282 (20.3)	322 (21.6)	<0.001
Smoking, n (%)	39 (36.1)	414 (29.8)	453 (30.3)	0.209
HF, n (%)	28 (25.9)	159 (11.5)	187 (12.5)	<0.001
AF, n (%)	3 (2.8)	16 (1.2)	19 (1.3)	0.315
OMI, n (%)	12 (11.1)	58 (4.2)	70 (4.7)	0.002
COPD, n (%)	2 (1.9)	9 (0.6)	11 (0.7)	0.410
Stroke, n (%)	8 (7.4)	65 (4.7)	73 (4.9)	0.302
PVD, n (%)	1 (0.9)	1 (0.1)	2 (0.1)	0.331

DM: diabetes mellitus; HF: heart failure; AF: atrial fibrillation; OMI: old myocardial infarction; COPD: chronic obstructive pulmonary disease; PVD: peripheral vascular disease; Data are presented as mean ± SD for continuous variables or n (%) for categorical variables.

### 3.2. Laboratory parameters and medication use

Laboratory findings revealed several significant differences between groups (Table 2). Patients in the death group had elevated fasting blood glucose levels (6.38 ± 3.42 vs. 5.73 ± 2.00 mmol/L, *P* = 0.004), higher serum creatinine (86.10 ± 76.94 vs. 70.10 ± 28.57 μmol/L, *P* < 0.001), and increased uric acid concentrations (321.37 ± 118.05 vs. 299.74 ± 86.10 μmol/L, *P* = 0.019), suggesting worse renal and metabolic status. In addition, left ventricular ejection fraction was significantly reduced in the death group (57.66 ± 11.21% vs. 62.92 ± 5.58%, *P* < 0.001), indicating poorer cardiac function. No significant differences were observed between the two groups in terms of lipid profiles, including total cholesterol, triglycerides, HDL-C, and LDL-C. Furthermore, the use of secondary prevention medications such as aspirin, clopidogrel, and statins was similar across groups (*P* > 0.05 for all), reflecting consistent adherence to post-PCI medication protocols.

**Table 2:** Laboratory parameters and medication use in patients.

	Death (n = 108)	Survival (n = 1387)	Total (n = 1495)	P values
Glu (mmol/L)	6.38 ± 3.42	5.73 ± 2.00	5.78 ± 2.14	0.004
Cr (umol/L)	86.10 ± 76.94	70.10 ± 28.57	71.25 ± 34.61	<0.001
UA (umol/L)	321.37 ± 118.05	299.74 ± 86.10	301.30 ± 88.93	0.019
BIL (umol/L)	10.05 ± 5.68	9.30 ± 5.13	9.35 ± 5.17	0.159
TC (mmol/L)	4.34 ± 0.95	4.31 ± 1.09	4.31 ± 1.08	0.765
TG (mmol/L)	1.89 ± 1.15	2.00 ± 1.51	1.99 ± 1.48	0.476
HDL-C (mmol/L)	1.08 ± 0.45	1.09 ± 0.32	1.09 ± 0.33	0.820
LDL-C (mmol/L)	2.82 ± 0.93	2.69 ± 0.96	2.7 ± 0.95	0.164
EF, (%)	57.66 ± 11.21	62.92 ± 5.58	62.49 ± 6.38	<0.001
Aspirin, n (%)	106(98.1%)	1370(98.8%)	1476 (98.7)	0.910
Clopidogrel, n (%)	101(93.5%)	1332(96.0%)	1433 (95.9)	0.334
Statin, n (%)	103(95.4%)	1296(93.4%)	1399 (93.6)	0.559

Glu: glucose; Cr: creatinine; UA: uric acid; BIL: bilirubin; TC: total cholesterol; TG: triglycerides; HDL-C: high-density lipoprotein cholesterol; LDL-C: low-density lipoprotein cholesterol; EF: Ejection Fraction. Data are expressed as mean ± SD or n (%), as appropriate.

### 3.3. Feature selection and multivariate analysis

**Fig 2:**
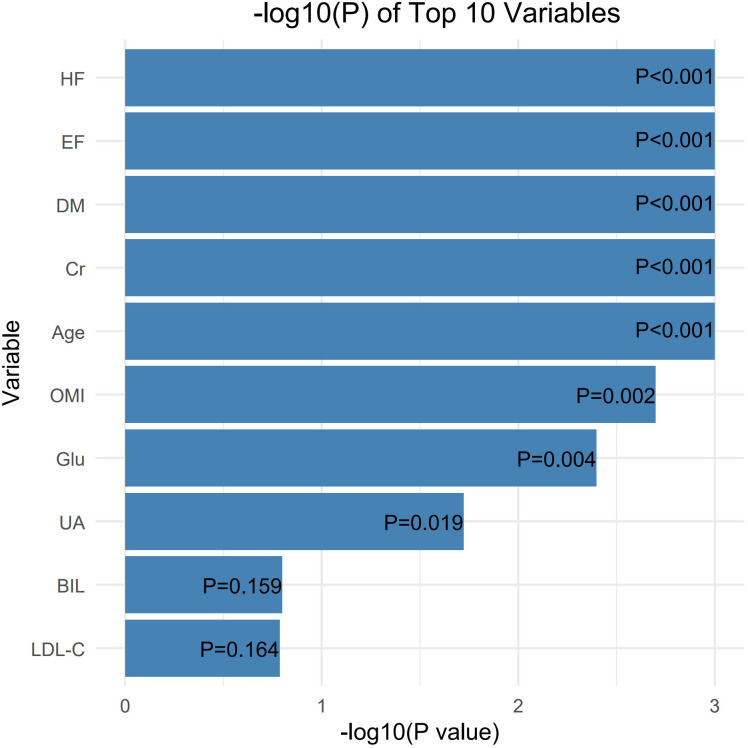
Top 10 clinical features identified by univariate analysis. Abbreviations: HF = heart failure; EF = ejection fraction; DM = diabetes mellitus; Cr = creatinine; OMI = old myocardial infarction; Glu = glucose; UA = uric acid; BIL = bilirubin; LDL-C = low-density lipoprotein cholesterol.

**Fig 3:**
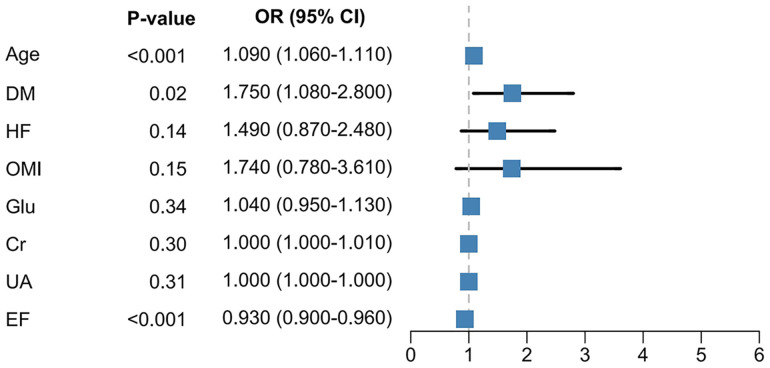
Forest plot of multivariate logistic regression analysis. Abbreviations: DM = diabetes mellitus; HF = heart failure; OMI = old myocardial infarction; Glu = glucose; Cr = creatinine; UA = uric acid; EF = ejection fraction.

To identify prognostic factors associated with all-cause mortality following PCI in patients with NSTE-ACS, univariate logistic regression was conducted across clinical and laboratory features. The top 10 variables ranked by statistical significance are shown in Fig 2, with corresponding *P* values as follows: heart failure (HF, *P* < 0.001), ejection fraction (EF, *P* < 0.001), diabetes mellitus (DM, *P* < 0.001), serum creatinine (Cr, *P* < 0.001), age (*P* < 0.001), old myocardial infarction (OMI, *P* = 0.002), fasting glucose (Glu, *P* = 0.004), uric acid (UA, *P* = 0.019), bilirubin (BIL, *P* = 0.159), and LDL-C (*P* = 0.164). Among them, eight variables with *P* < 0.05 were considered significant and included in the subsequent multivariate logistic regression analysis for further evaluation. In the multivariate model (Fig 3), three variables were independently associated with an increased risk of all-cause mortality. Age was positively associated with mortality (OR = 1.090, 95% CI: 1.060 to 1.110, *P* < 0.001), as was the presence of diabetes mellitus (OR = 1.750, 95% CI: 1.080 to 2.800, *P* = 0.020). In contrast, a higher ejection fraction was inversely associated with risk (OR = 0.930, 95% CI: 0.900 to 0.960, *P* < 0.001), indicating the protective role of preserved cardiac function. Other variables such as HF, OMI, Cr, Glu, and UA showed attenuated statistical associations after adjustment, suggesting that their effects may be confounded or partially mediated by other covariates. Age, DM, and EF were ultimately retained as the most stable and informative predictors for subsequent model construction.

### 3.4. Model evaluation and comparison

**Fig 4:**
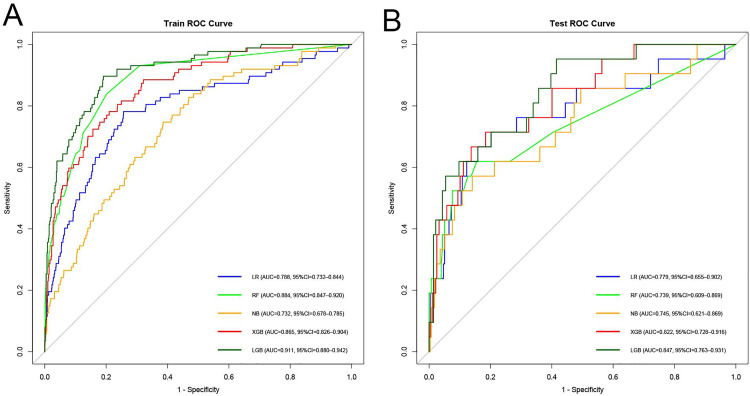
ROC curves of the predictive nomogram for PCI. (A) Training set; (B) Validation set. Abbreviations: LR = logistic regression; RF = random forest; NB = naive Bayes; XGB = extreme gradient boosting; LGB = light gradient boosting machine.

Five machine learning models, including logistic regression (LR), random forest (RF), XGBoost (XGB), LightGBM (LGB), and Naïve Bayes (NB), were developed using eight selected features to predict all-cause mortality following PCI in patients with NSTE-ACS. Model discrimination was evaluated using receiver operating characteristic (ROC) curves and the area under the curve (AUC). In the training cohort (Fig 4A), LightGBM exhibited the highest discriminatory ability with an AUC of 0.911 (95% CI: 0.880 to 0.942), followed by random forest (AUC = 0.884, 95% CI: 0.847 to 0.920), XGBoost (AUC = 0.865, 95% CI: 0.826 to 0.904), and logistic regression (AUC = 0.788, 95% CI: 0.732 to 0.844). Naïve Bayes had the lowest AUC in the training cohort, with a value of 0.732 (95% CI: 0.678 to 0.785), though still above the acceptable threshold of 0.7. In the independent test cohort (Fig 4B), LightGBM again achieved the highest AUC (0.847, 95% CI: 0.763 to 0.931), followed by XGBoost (AUC = 0.822, 95% CI: 0.728 to 0.916), logistic regression (AUC = 0.779, 95% CI: 0.655 to 0.902), Naïve Bayes (AUC = 0.745, 95% CI: 0.621 to 0.869), and random forest (AUC = 0.739, 95% CI: 0.609 to 0.869). These findings suggest that all five models demonstrated acceptable discriminatory power, with LGB and XGB achieving consistent top-tier performance across both datasets.

### 3.5. LightGBM and XGBoost show superior calibration performance

**Fig 5:**
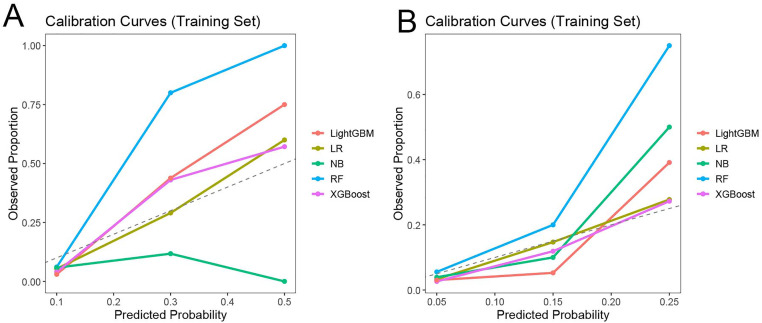
Calibration curves for assessing the accuracy of the predictive nomogram for PCI. (A) Training set; (B) Validation set. Abbreviations: LR = logistic regression; RF = random forest; NB = naive Bayes; XGB = extreme gradient boosting; LGB = light gradient boosting machine.

Model calibration was assessed using calibration curves. In the training cohort (Fig 5A), LightGBM and XGBoost exhibited calibration curves that closely approximated the ideal diagonal line, indicating good agreement between predicted and observed risks. Logistic regression also showed acceptable calibration, while random forest tended to overestimate risk at higher probability ranges. Naïve Bayes displayed notable deviation from the ideal line. Similar trends were observed in the test cohort (Fig 5B), where LightGBM and XGBoost again demonstrated favorable calibration, reinforcing their reliability for clinical prediction. Taken together, these results indicate that LightGBM and XGBoost outperformed other models in both discrimination and calibration, supporting their potential utility for individualized risk prediction in NSTE-ACS patients after PCI.

## 4. Discussion

This study developed and evaluated five supervised machine learning models to predict all-cause mortality in patients with non–ST-segment elevation acute coronary syndrome (NSTE-ACS) undergoing percutaneous coronary intervention (PCI), based on a real-world cohort of 1,495 patients. Using eight routinely available clinical variables, we found that age, diabetes mellitus (DM), and ejection fraction (EF) were independently associated with mortality. Among the five models tested, LightGBM and XGBoost consistently demonstrated superior predictive performance in both discrimination and calibration. These findings underscore the feasibility and potential clinical value of machine learning approaches in improving individualized risk assessment in NSTE-ACS populations.

Age, EF, and DM emerged as the most informative and stable predictors of all-cause mortality in our multivariate logistic regression model, which aligns with the body of evidence supporting their prognostic importance. Advanced age has long been associated with worse cardiovascular outcomes due to cumulative vascular injury, increased comorbidity burden, and impaired physiologic reserve [18,19]. Reduced EF is a marker of impaired myocardial function and has been strongly linked to adverse events post-PCI in both ST-elevation and non–ST-elevation ACS cohorts [20]. Diabetes, as a systemic metabolic disorder, contributes to endothelial dysfunction, accelerated atherosclerosis, and impaired collateral circulation, leading to worse post-revascularization outcomes [21,22]. The identification of these factors supports the clinical validity of the feature set used in our machine learning models.

Traditional risk stratification tools such as the GRACE and TIMI scores have been widely used to guide clinical decisions in ACS [3,6]. However, these scores have limitations, including reliance on predefined variables, linear risk weighting, and decreasing calibration accuracy in modern clinical practice. Machine learning techniques can overcome many of these challenges by modeling complex nonlinear interactions and incorporating a broader range of data features [23,24]. In our study, the application of LightGBM and XGBoost yielded higher area under the curve (AUC) values in both training and test datasets compared with logistic regression, suggesting that these algorithms provide more accurate risk discrimination. In particular, LightGBM achieved an AUC of 0.847 in the test cohort, outperforming random forest, naïve Bayes, and logistic regression. This is consistent with prior studies showing that gradient boosting models often outperform other machine learning approaches in structured clinical datasets [25,26].

In addition to discrimination, model calibration is crucial for clinical adoption, as it reflects how well predicted probabilities match actual observed outcomes. Poor calibration can lead to misinformed clinical decisions, especially at the extremes of risk. In our analysis, both LightGBM and XGBoost demonstrated favorable calibration, with predicted probabilities closely aligning with observed event rates. This performance supports their potential use in clinical settings where precise risk estimation is critical. Logistic regression showed acceptable but inferior calibration, while naïve Bayes substantially deviated from the ideal calibration curve, likely due to its assumption of conditional independence among features [27].

Few prior studies have specifically focused on NSTE-ACS, which represents a clinically heterogeneous population with variable ischemic burden and subtler ECG changes. Most machine learning studies in ACS have either combined STEMI and NSTE-ACS cohorts or targeted major adverse cardiovascular events (MACE) rather than all-cause mortality. However, these models lacked external validation and did not specifically assess long-term mortality. Our study, by contrast, focused solely on NSTE-ACS patients, used a clearly defined and clinically relevant endpoint, and compared five modeling strategies using both discrimination and calibration metrics. These strengths enhance the credibility and practical relevance of our findings.

Nevertheless, several limitations should be acknowledged. First, this study is based on a retrospective analysis of a single-center cohort, and although internal validation was performed, external validation in diverse populations is needed. Second, we focused only on all-cause mortality, and did not analyze other important clinical endpoints such as cardiovascular death, reinfarction, or bleeding events. Third, baseline troponin, creatine kinase, and NT-proBNP values were available only for a subset of patients, but not for the entire cohort. Because of the considerable proportion of missing values, inclusion of these biomarkers in the analysis would have introduced selection bias; therefore, they were not incorporated into the primary analysis, which should be considered as a study limitation. Fourth, the black-box nature of some machine learning models can hinder clinical interpretability and trust. While methods such as SHAP (Shapley Additive Explanations) can help elucidate feature contributions, further work is required to enhance transparency and user acceptance. Lastly, as clinical practice evolves, models will need to be periodically retrained to maintain performance.

In summary, our study contributes to the growing body of evidence supporting machine learning as a powerful tool for cardiovascular risk stratification. By demonstrating the superiority of LightGBM and XGBoost in predicting mortality among NSTE-ACS patients post-PCI, we provide a foundation for future work on deploying these models in clinical workflows. Future studies should aim to validate these findings in external cohorts.

## 5. Conclusion

In this study of patients with NSTE-ACS undergoing PCI, we identified age, diabetes mellitus, and ejection fraction as independent predictors of all-cause mortality. Machine learning models, particularly LightGBM and XGBoost, outperformed traditional logistic regression and other algorithms in both discrimination and calibration. These findings highlight the potential of gradient boosting techniques to enhance individualized risk prediction and inform post-PCI management strategies. Further validation and implementation studies are warranted to translate these models into clinical decision-making tools.
